# Novel Squaramides and Squaramates Containing a Five-Membered Heterocyclic Ring: Synthesis, Structure, and Cytotoxicity

**DOI:** 10.3390/ijms27136047

**Published:** 2026-07-06

**Authors:** Georgi Tirolski, Boris Vasilev, Mariyana Atanasova, Georgi Momekov, Hristina Sbirkova-Dimitrova, Adriana Bakalova, Emiliya Cherneva

**Affiliations:** 1 Department of Pharmacology, Pharmacotherapy and Toxicology, Faculty of Pharmacy, Medical University of Sofia, Dunav 2 Street, 1000 Sofia, Bulgaria; g_tirolski@abv.bg (G.T.); 120588@students.mu-sofia.bg (B.V.); gmomekov@pharmfac.mu-sofia.bg (G.M.); 2 Institute of Organic Chemistry with Centre of Phytochemistry, Bulgarian Academy of Sciences, Acad. G. Bonchev Str., Build. 9, 1113 Sofia, Bulgaria; 3 Department of Chemistry, Faculty of Pharmacy, Medical University of Sofia, Dunav 2 Street, 1000 Sofia, Bulgaria; matanasova@pharmfac.mu-sofia.bg; 4 Centre of Excellence in Informatics and Information and Communication Technologies, 1113 Sofia, Bulgaria; 5 Institute of Mineralogy and Crystallography “Acad. Ivan Kostov”, Bulgarian Academy of Sciences, Acad. G. Bonchev Str., Build. 107, 1113 Sofia, Bulgaria; hsbirkova@gmail.com; 6 Center of Competence PERIMED-2, Vasil Aprilov Blvd. 15A, 4002 Plovdiv, Bulgaria

**Keywords:** squaric acid, squaramide, squaramate, Navarixin, cytotoxic activity

## Abstract

With the introduction of Navarixin in clinical trials, the role of squaric acid derivatives as bioisosteres gained popularity. Because of their distinctive electronic properties and hydrogen-bonding capacity, these compounds hold considerable promise for medicinal chemistry applications. In this study, a series of novel furan- and thiophene-containing squaric acid derivatives was synthesized via base-catalyzed nucleophilic substitution and characterized by spectroscopic techniques. The structures of three compounds were additionally confirmed by X-ray crystallography. Density functional theory calculations showed good agreement with the experimental vibrational spectra. In silico evaluation predicted favorable drug-like characteristics, including compliance with Lipinski’s rule of five and high gastrointestinal absorption. The cytotoxic activity of the synthesized compounds was assessed against HeLa, HT-29, HL-60, A-549, and MCF-7 cancer cell lines, as well as the non-cancerous CCL-1 cell line. Several derivatives displayed moderate to strong antiproliferative activity with selectivity toward malignant cells. Compound **3d** exhibited the most pronounced improvement (five-fold) over Navarixin in HL-60 cells 5.81 µM, while compounds **3a** and **3c** demonstrated superior potency and selectivity in A-549 cells (10.33 µM and 9.65 µM). These findings identify squaric acid derivatives as promising candidates for further anticancer drug development and structure–activity relationship studies.

## 1. Introduction

Squaric acid (3,4-dihydroxycyclobut-3-ene-1,2-dione, SQ) is a highly strained, aromatic four-membered carbocyclic system characterized by reduced electron density and pronounced polarization. Its unique electronic structure arises from the presence of two acidic hydroxyl groups and two strongly electron-withdrawing carbonyl groups. Within this framework, the hydroxyl groups act as hydrogen-bond donors, while the carbonyl oxygens function as proton acceptors, enabling SQ and its derivatives to participate in hydrogen-bonding interactions [1,2]. These properties make the squaric core an attractive scaffold in medicinal chemistry. Among SQ derivatives, squaramates and squaramides represent the most important and widely studied classes. Squaramates, formerly referred to as esteramides, and squaramides, which may be symmetric or asymmetric diamides, are considered valuable bioisosteres of urea and peptides [3,4]. They offer enhanced rigidity, well-defined hydrogen-bonding patterns, and favorable physicochemical properties. As a result, these compounds have been increasingly incorporated into drug design and have demonstrated promising biological activity across multiple therapeutic areas [5,6,7].

In recent years, SQ derivatives have gained relevance in oncology, theranostics, and the treatment of infectious diseases. Several squaramide-based compounds have shown activity against *Mycobacterium tuberculosis*. In this case, novel compounds have emerged as a new class of antimycobacterial agents targeting ATP synthase, interacting with distinct binding sites, which allows them to overcome existing resistance mechanisms while maintaining strong activity and reduced toxicity [8,9,10]. Also, squaramide-based compounds have demonstrated promising antiparasitic activity, with improved selectivity indices and lower toxicity compared to standard treatments such as benznidazole. Their favorable pharmacokinetic properties, compliance with Lipinski’s rule of five, and relatively simple synthesis support their potential as accessible therapeutic agents for neglected tropical diseases such as leishmaniasis and Chagas disease [11,12]. Squaric acid derivatives have also attracted considerable interest in theranostics, particularly in nuclear medicine [13]. In this context, SQ diesters enable easy conjugation of bifunctional chelators such as DOTA, NOTA, and AAZTA to target vectors including peptides, small molecules, and monoclonal antibodies under mild conditions. This strategy avoids many of the limitations associated with conventional coupling methods, such as side-product formation, harsh reaction conditions, and the need for protecting groups, while maintaining favorable biological activity and pharmacokinetics [14,15,16]. 

Squaric acid dibutyl ester (SADBE) is a topical immunotherapeutic agent used in alopecia areata due to its favorable safety profile and low systemic toxicity. It promotes hair regrowth by inducing a localized inflammatory response that shifts hair follicles from the telogen to the anagen phase. Beyond hair disorders, SADBE has also been investigated for the treatment of herpes labialis, involving T cells. Clinical evidence suggests that SADBE can prolong the interval between outbreaks and reduce disease recurrence, highlighting its broader immunomodulatory potential [17,18,19].

**Figure 1:**
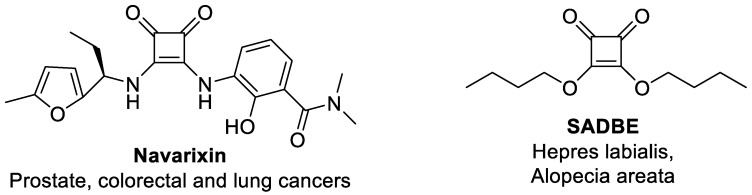
Notable SQ derivatives in clinical trials.

Within this context, *Navarixin* (Nav) has emerged as the most important squaramide-based drug candidate. Initially developed as a potent and selective antagonist of the CXC chemokine receptor 2 (CXCR2) for asthma and psoriasis, *Navarixin* has since been shown to exert a broader spectrum of biological effects, including modulation of tumor plasticity, inhibition of tumor growth and colony formation, and induction of apoptosis [20,21]. Nav inhibits CXCR2 signaling in a balanced manner, affecting both G protein activation and β-arrestin recruitment without bias toward either pathway. As a small-molecule antagonist of CXCR1 and CXCR2, *Navarixin* has demonstrated therapeutic potential across multiple solid tumor types by disrupting CXCR1/2-driven signaling networks. Preclinical studies further suggest that CXCR2 inhibition can suppress tumor progression, reduce lineage plasticity, and enhance the efficacy of immune checkpoint blockade. These findings have led to clinical investigation, including a phase II trial (NCT03473925) evaluating *Navarixin* in combination with Pembrolizumab in patients with advanced or metastatic castration-resistant prostate cancer (CRPC), microsatellite-stable colorectal cancer (MSS CRC), and non-small-cell lung cancer (NSCLC) [22]. Collectively, these results show the therapeutic potential of squaramide-containing compounds and support the continued development of structurally related analogues (Figure 1).

With these considerations, we report herein the design, synthesis, and in vitro evaluation of five new squaric acid derivatives, comprising three squaramates and two squaramides. The compounds incorporate five-membered heteroaromatic rings, namely furan and thiophene. These heterocyclic moieties were selected to resemble one of the substituents present in *Navarixin* while allowing systematic variation of electronic and steric properties. The resulting compounds represent structurally simplified analogues of *Navarixin* and provide a basis for further investigation of structure–activity relationships within this class of SQ derivatives.

## 2. Results and Discussion

### 2.1. Design and In-Silico Evaluation

A series of five squaramides and squaramates containing a five-membered heterocyclic ring was designed by combining SQ esters with various amines. The selected derivatives incorporate molecular fragments found in numerous biologically active compounds, which are essential for their functional activity (Table 1).

Prior to the synthesis of the SQ derivatives, a comprehensive in silico profiling was undertaken to support compound prioritization. This evaluation encompassed key aspects of drug-likeness, physicochemical properties, and ADME behavior (absorption, distribution, metabolism, and excretion), with the aim of identifying candidates with favorable developability characteristics. The analysis was performed using the SwissADME web platform (https://www.swissadme.ch/, accessed on 3 February 2026) alongside the ACD/Labs Suite v. 9.10 (Advanced Chemistry Development, Inc., Toronto, ON, Canada). In addition, pharmacokinetic parameters were estimated using previously validated quantitative structure–pharmacokinetics relationship (QSPkR) models [23,24,25], enabling further insight into the expected in vivo behavior of the compounds. The outcomes of this integrated computational assessment are summarized in Table 2.

The molecular weight distribution of the derivatives (221.21–304.38 g/mol) places this series firmly within the lead-like chemical space rather than that of fully optimized drug-like molecules [26]. In terms of ionization behavior, the compounds display weakly basic characteristics, with predicted pKa values spanning from −2.01 to −0.53. Consequently, under physiological conditions (pH 7.4), they are expected to remain predominantly in their neutral form, with a negligible fraction present as ionized species (f_b_ = 0). This neutral character is likely to favor passive membrane permeability and may facilitate intracellular access to relevant biological targets. 

The consensus LogP values for the designed derivatives span from 0.84 to 2.24, indicating an overall low-to-moderate lipophilicity across the series. Compounds **3a**–**3c** and **3e** exhibit LogP values close to 1 (0.84–1.40), consistent with a well-balanced hydrophilic–lipophilic profile, whereas derivatives **3e** (LogP = 2.24) shifted toward higher, yet still moderate lipophilicity. This lipophilicity window is well aligned with medicinal chemistry design principles, as it reflects a desirable compromise between hydrophilicity and lipophilicity. Such a balance is critical for maintaining adequate aqueous solubility while still allowing effective partitioning into lipid bilayers, thereby facilitating both proper dissolution and passive transmembrane transport. From a structural perspective, compounds **3a**–**3c** contain a single five-membered heterocyclic ring, which is consistent with their lower LogP values. In contrast, derivatives **3d** and **3e** incorporate two such heterocyclic systems. In the case of the thiophene-containing analogue (**3d**), this results in a moderate increase in lipophilicity, whereas for the furan-containing derivative (**3e**), the LogP remains below 1. This difference can be attributed to the presence of the oxygen atom in the furan ring, which enhances polarity and enables participation in hydrogen bonding with surrounding water molecules, thereby promoting a more hydrophilic character and improved aqueous solubility. Within the **3a**–**3c** subset, the nature of the R_1_ substituent appears to play a less pronounced influence on lipophilicity compared to the heterocyclic ring (R_2_). Specifically, methyl substitution (methoxy derivatives **3a** and **3b**; LogP = 1.13 and 1.40) leads to slightly higher lipophilicity compared to ethyl substitution (ethoxy derivative **3c**; LogP = 0.84), although the overall effect remains modest. This modest trend may tentatively be attributed to subtle steric and solvation effects that influence the ability of the oxygen to interact with the aqueous environment, although the overall impact remains limited. Finally, the influence of alkyl chain length is evident when comparing homologous pairs such as **3a** and **3b**, where the addition of a methylene unit results in an expected increase in LogP of approximately 0.3, consistent with established trends in homologous series. 

The calculated polar surface area (PSA) values span from 59.75 to 114.68 Å^2^, reflecting a moderate polarity across the series. The highest PSA value is observed for compound **3d** (114.68 Å^2^), which incorporate two five-membered heterocyclic rings, thereby increasing the overall polar surface. In contrast, compound **3e**, despite also containing two heterocyclic rings, exhibits a lower PSA value (84.48 Å^2^), reflecting the bioisosteric influence of the furan oxygen, which modulates polarity differently compared to the thiophene analogue. Intermediate PSA values (83.84 Å^2^) are found for analogues **3a** and **3b**, characterized by a methoxy substituent at the R_1_ position and a thiophene ring at R_2_. Importantly, the entire PSA range falls within the threshold typically associated with favorable oral bioavailability (PSA ≤ 140 Å^2^), suggesting that these compounds are likely to retain adequate membrane permeability and high intestinal absorption (>90%) [27,28,29,30]. Furthermore, four derivatives lie within the PSA window commonly linked to central nervous system exposure (PSA < 60–90 Å^2^), indicating a potential capacity for blood–brain barrier penetration [31,32]. 

The compounds exhibit a moderate degree of conformational flexibility, with four to six rotatable bonds across the series—four in **3a**, five in **3b**–**3c**, and up to six in **3d** and **3e**. In the latter analogues, however, this flexibility is effectively constrained by extended π-conjugation spanning the multiple aromatic rings and connecting linkers, resulting in a more conformationally biased and relatively rigid framework. In addition, the molecules present a balanced hydrogen-bonding profile, comprising one or two hydrogen bond donors and two to four acceptors. Together with their favorable molecular weight and lipophilicity, these features ensure compliance with Lipinski’s rule of five, supporting their suitability as orally bioavailable candidates [26]. 

From an ADME perspective, the SQ derivatives display an overall favorable developability profile. Most compounds are predicted to be readily soluble in aqueous media, apart from **3d** which falls within the moderately soluble range, likely reflecting its comparatively higher lipophilicity. All derivatives exhibit high predicted gastrointestinal (GI) absorption, supported by bioavailability scores in the range of 0.55–0.85, indicating a reasonable likelihood of achieving measurable oral exposure (>10% in rats). This behavior is consistent, in part, with the low fraction of sp^3^-hybridized carbons—particularly in **3d** and **3e**—a feature often associated with acceptable oral performance at early stages of drug discovery. In terms of distribution, most of the compounds are predicted to have limited permeability across the blood–brain barrier. The predicted metabolic profile suggests that all derivatives may inhibit CYP1A2, while CYP2C19 inhibition is anticipated for **3b** and **3d**. Notably, the latter compound, **3d**, is also predicted to inhibit CYP2C9 and CYP3A4, indicating a broader potential for metabolic interactions within this subset. None of the compounds are predicted to be substrates of P-glycoprotein, which may favor intracellular retention and reduce the likelihood of efflux-mediated resistance. 

Overall, the series aligns closely with lead-like design principles, with molecular weights largely within the 250–350 g/mol range (with **3a**–**3d** slightly below this window), XLogP values generally not exceeding 3.5, and a limited number of rotatable bonds (≤7) [33]. Finally, synthetic accessibility scores ranging from 2.58 to 2.94 indicate that these derivatives are readily tractable from a synthetic standpoint, supporting their suitability for further optimization and structure–activity relationship exploration.

With respect to the predicted pharmacokinetic parameters [34,35], the predicted steady-state volumes of distribution (VDss = 0.82–2.13 L/kg) indicate that all compounds distribute well beyond the vascular compartment. When considered in the context of physiological fluid volumes—approximately 0.2 L/kg for extracellular fluid and 0.6–0.7 L/kg for total body water [36]—these values are consistent with moderate to extensive tissue partitioning across the series. Compound **3c**, with VDss value below 1 L/kg, display a more balanced distribution profile, suggesting partial retention within plasma and extracellular fluids. In contrast, derivatives **3d** and **3e** (VDss > 1.5 L/kg) show pronounced tissue uptake, indicative of enhanced partitioning into peripheral compartments, likely driven by increased lipophilicity and expanded aromatic surface area. This behavior is consistent with the well-established distribution patterns of weakly basic compounds, for which a substantial fraction of the apparent volume of distribution is attributed to sequestration within lipid-rich tissues. Such compounds typically exhibit strong affinity for phospholipid membranes through electrostatic interactions between their protonated centers and anionic head groups, as well as binding to plasma proteins such as α_1_-acid glycoprotein and, to a lesser extent, human serum albumin. In addition, lysosomal ion trapping may further contribute to intracellular accumulation, collectively promoting extensive tissue distribution and elevated VDss values. The predicted systemic clearance values (3.699–5.076 mL/min/kg) indicate a low-to-moderate elimination profile across the series, suggesting that rapid metabolic turnover is unlikely to be a limiting factor. In contrast, the observed variation in half-life (t_1/2_ = 1.91–6.73 h) appears to be primarily governed by differences in volume of distribution and plasma protein binding rather than clearance alone. Compound **3d** exhibits the longest half-life, consistent with its low unbound fraction (f_u_ = 0.084) and high VDss, indicative of extensive tissue partitioning and potential sequestration in lipid-rich compartments. A similar, though less pronounced, trend is observed for **3e**. In contrast, compounds **3a**–**3c** and **3e**, which display higher f_u_ values and lower VDss, are characterized by shorter half-lives, reflecting a greater proportion of freely circulating drug and reduced tissue retention. These relationships highlight the interplay between lipophilicity, protein binding, and distribution in dictating systemic exposure and underscoring the potential to fine-tune pharmacokinetic behavior within this scaffold through targeted structural modification.

Overall, the integrated physicochemical, ADME, and pharmacokinetic profiling highlights this series as a well-balanced set of lead-like candidates with tunable properties. The combination of low molecular weight, predominantly neutral character at physiological pH, and controlled lipophilicity supports a favorable balance between aqueous solubility and membrane permeability. At the same time, the divergence observed for **3d** and **3e** underscores the sensitivity of both lipophilicity and polarity to subtle heterocyclic modifications, emphasizing the importance of bioisosteric design in fine-tuning molecular properties. The predicted distribution and binding profiles further indicate that systemic exposure is primarily controlled by the interplay between tissue partitioning and plasma protein binding, rather than clearance alone. Collectively, these findings position the SQ scaffold as a tractable and chemically versatile platform, where targeted structural modifications can be effectively leveraged to optimize both pharmacokinetic performance and overall developability, thereby supporting its continued exploration in lead optimization efforts.

### 2.2. Synthesis

**Scheme 1:**
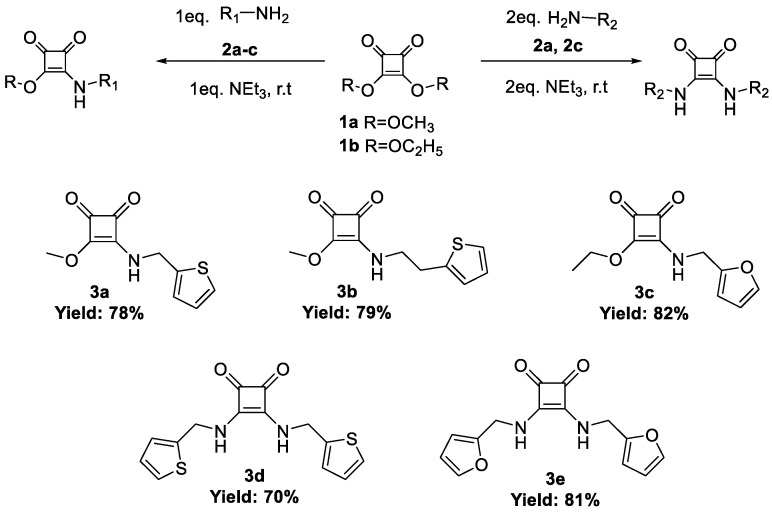
Synthetic scheme for squaramates and squaramides **3a**–**3e**.

Compounds **3a**–**3e** were synthesized via base-catalyzed nucleophilic substitution of methyl or ethyl squarate with the corresponding amines. Mono- or bis-aminated SQ derivatives were obtained depending on the amine stoichiometry (Scheme 1). Reaction of methyl or ethyl squaric acid ester with the appropriate heteroarylalkyl amine under mild conditions afforded the squaramide products in moderate to good yields. The synthesis is described by Tietze [37]. The structures of the target compounds were identified based on IR and NMR spectra and melting points were obtained. X-ray analysis was used to confirm the structures of three synthesized compounds.

### 2.3. Spectroscopy

The experimental IR spectra show characteristic vibrational bands for the studied functional groups. The carbonyl stretching modes ν^s^_C=O_ and ν^as^_C=O_ are observed in the ranges 1793–1799 cm^−1^ and 1715–1642 cm^−1^, respectively, with only minor variations between compounds. Similarly, the C=C stretching vibrations appear around 1570–1620 cm^−1^, again with small shifts depending on the molecular environment. The stretching vibration of secondary amine is of great importance during preparation of the compounds—one criterium for reaction completion is loss of ν^s^ and ν^as^ vibrations of the precursor amines. The resulting squaramato/squaramido NH stretching bands are detected at lower frequencies (3145–3153 cm^−1^), indicating the influence of intermolecular hydrogen bonding interactions which weaken the vibrational frequency of N-H. The DFT calculated vibrational frequencies correspond well with the experimental data for most functional groups, particularly for the C=O and C=C stretching modes, where only minor deviations are observed. These small differences are expected due to the approximations inherent in theoretical methods. A more pronounced difference is found for the NH stretching vibrations, where the calculated values (3470–3478 cm^−1^) are consistently higher than the experimental ones. This deviation arises because the calculations were performed in the gas phase and therefore do not account for hydrogen bonding effects present in the solid state.

NMR spectra are consistent with the squaramates/squaramides structure and display the expected features for N-H, heteroaromatic, and aliphatic fragments. ^1^H NMR spectra show signals in the downfield region (8.5–9.5 ppm) for N-H protons. These signals are typically broad and weak due to hydrogen bonding. The aromatic region (6–8 ppm) corresponds to protons of thiophene or furan, which appear as multiplets. In the aliphatic region, methylene (N-CH_2_-Ar) protons consistently register at 4.5–5.0 ppm. Terminal methyl (CH_3_) groups, as in **3c** generally appear below 2 ppm. An important distinction can be observed in the NMR spectra: signals corresponding to ester aliphatic protons are absent in squaramides, whereas they are present from 4.2–4.9 ppm, as expected, in squaramates. ^13^C NMR spectra show characteristic carbonyl and double bond resonances for the squaric fragment in 160–190 ppm region. Signals corresponding to aromatic carbons are observed in the expected regions 100–160 ppm, while aliphatic carbons (observed in methoxy and ethoxy groups in squaramates and methyl-amino group of squaramides) appear at lower ppm. In symmetrical derivatives, a reduced number of carbon signals is observed, which corresponds to equivalent environments.

### 2.4. X-Ray Crystal Structure Analysis

**Figure 2:**
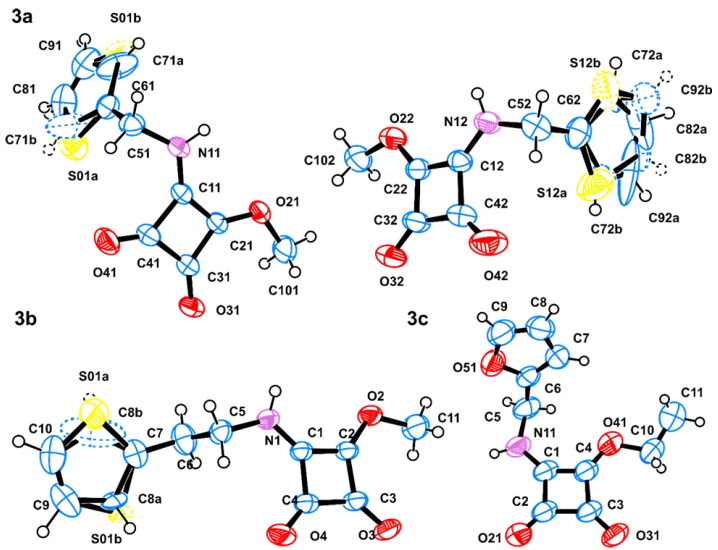
Molecular structures of compounds **3a**–**3c** shown as ORTEP representations with displacement ellipsoids drawn at the 50% probability level. Hydrogen atoms are shown as spheres of arbitrary radius, and disorder (where present) is depicted over two positions.

Single-crystal analysis reveals that compounds **3a**, **3b**, and **3c** crystallize in a centrosymmetric manner. **3a** and **3b** crystallize in the monoclinic system, belonging to space groups Pc and P2_1_/n, respectively, whereas **3c** forms a triclinic lattice with space group P-1 (Figure 2). The asymmetric unit of **3a** contains two crystallographically independent molecules, while one molecule is present in the asymmetric units of compounds **3b** and **3c**. 

The molecular structures of compounds **3a**, **3b**, and **3c** were determined by single-crystal X-ray diffraction, and the corresponding crystallographic data are presented in Table S1. In all cases, the molecules adopt the expected squaramide/squaramate architecture, built around a four-membered ring that remains essentially planar, consistent with effective π-electron delocalization, and corresponding to this class of compounds. This is reflected in the observed C-N and C=O bond distances, which fall within the typical ranges (Table S1). Although the overall molecular motif is conserved, the three compounds differ in their crystallographic symmetry and conformational details: **3a** and **3b** crystallize in the monoclinic system (space groups Pc and P2_1_/n, respectively), while **3c** adopts a triclinic lattice (P-1). Notably, the asymmetric unit of **3a** contains two crystallographically independent molecules, which display similar geometries but differ slightly in the orientation of their substituents. Another point of distinction concerns the peripheral substituents—**3a** and **3b** show clear evidence of positional disorder involving the sulfur-containing fragments, distributed over two orientations with partial occupancies, whereas **3c** appears fully ordered.

From a supramolecular perspective, the packing in all three structures is primarily governed by intermolecular N-H···O hydrogen bonds involving the squaramide NH groups and carbonyl oxygen atoms, in agreement with the tendency of squaramides to act as strong hydrogen-bond donors and acceptors. In **3a**, these interactions are close to linear and give rise to extended hydrogen-bonded chains, further reinforced by a network of weaker C-H···O and C-H···S contacts. A similar model pattern is observed in **3b**, where a single dominant N-H···O interaction is accompanied by secondary contacts that appear to play a supporting role in stabilizing the lattice. In contrast, **3c** forms a more discrete hydrogen-bonding motif, which, together with the absence of disorder and the lower symmetry, leads to a more clearly defined packing arrangement. These observations suggest that subtle differences in substituent flexibility and solid-state organization may influence not only the crystal architecture but also properties relevant to molecular recognition and interaction with biological targets, which could be reflected in the observed cytotoxic activity of the compounds.

### 2.5. Optimized Geometry

**Figure 3:**
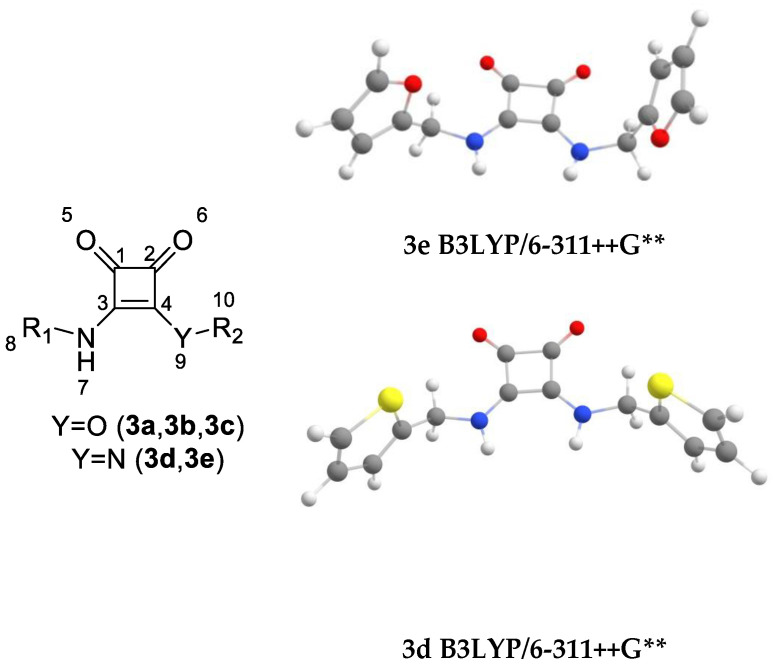
Structure of SQ derivatives with numbered atoms and optimized structures.

The use of experimental analytical methods in combination with quantum-chemical calculations is a suitable approach for obtaining structural information in cases where it is impossible to grow single crystals for X-ray diffraction analysis (Figure 3). They also provide information on physical quantities such as dipole moment, geometric parameters, charges, spectral characteristics, and others [38,39,40].

As for bond lengths, the theoretical (DFT) values are slightly larger than the experimental ones in most cases. This is particularly noticeable for the C3–N7 bond, where DFT overestimates the length by about 0.01–0.03 Å. The C3–C4 bond lengths match very closely, differing only marginally (=0.002–0.005 Å). Regarding bond angles, DFT consistently predicts slightly larger C3N7R8 angles than those observed experimentally, with differences of about 0.5–1.5° and <1° for the C4Y9R10 angles (Table 3). Overall, the DFT method reproduces the experimental geometry well, with small systematic overestimations in bond lengths and slight variations in bond angles. This confirms its reliability for describing the molecular structure of the compounds.

The relative Gibbs free energy differences (ΔG) between the conformers, where the N-H hydrogen is oriented toward the carbonyl groups (conformer A) or directed away from them (conformer B), was calculated using the same DFT methods. For compounds **3a** (ΔG = −0.13 kJ/mol, conformer B) and **3b** (ΔG = −0.20 kJ/mol, conformer A), the energy differences are negligible, which suggests that both forms are thermodynamically equivalent and can coexist at room temperature. In the case of **3c** (ΔG = 1.90 kJ/mol, conformer A), the small energy difference indicates only a slight preference, consistent with a nearly balanced population. Both **3d** (ΔG = 8.27 kJ/mol, conformer B) and **3e** (ΔG = 11.7 kJ/mol, conformer B) show reduced stability, and a limited contribution to the conformational structures.

### 2.6. Cytotoxicity

The cytotoxic activity of the synthesized squaramide analogues was evaluated against a panel of human cancer cell lines, including HeLa, HT-29, HL-60, A-549, and MCF-7, as well as the non-cancerous CCL-1 fibroblast cell line, using a modified MTT assay. Navarixin (Nav) was used as a reference compound. The obtained IC_50_ and selectivity index (SI) values are presented in Table 4. Overall, the investigated compounds displayed moderate activity with differences in both potency and selectivity depending on the cancer cell type. 

No significant activity was observed against HeLa cells, as all compounds exhibit IC_50_ values above 100 μM. Among the tested derivatives, **3a**, **3c**, and **3d** demonstrated the best cytotoxic profiles. Compounds **3a** and **3c** were particularly active against A-549 cells, exhibiting IC_50_ values of 10.33 and 9.65 μM, respectively. These values corresponded to the highest selectivity indices in the series (SI = 17.73 and 19.73, respectively), indicating a marked preference for cancer cells. Additionally, **3a** showed acceptable activity against MCF-7 cells (IC_50_ = 23.87 μM), suggesting a broader spectrum of activity. The same goes for compound **3c** with cytotoxicity toward HL-60 (IC_50_ = 26.78 μM, SI = 7.11), HT-29 (IC_50_ = 24.95 μM, SI = 7.63), and MCF-7 cells (IC_50_ = 34.26 μM, SI = 5.56). The most potent analogue of the whole series is **3d**, with remarkable activity against HL-60 cells, an IC_50_ value of 5.81 μM, and the highest selectivity index observed among all tested compounds (SI = 29.07). In addition, **3d** showed good activity toward MCF-7 cells (IC_50_ = 15.74 μM, SI = 10.73) and HT-29 cells (IC_50_ = 20.44 μM, SI = 8.27), indicating a highly favorable therapeutic window. Importantly, all synthesized compounds exhibited relatively low toxicity toward the non-cancerous CCL-1 cell line, with IC_50_ values ranging from 168.90 to 197.33 μM. 

Comparison with Navarixin revealed several notable differences. While Navarixin showed moderate activity against HL-60 (IC_50_ = 29.10 μM, SI = 5.30) and A-549 cells (IC_50_ = 24.11 μM, SI = 6.39), it was substantially less potent against HT-29 and MCF-7 cells, with IC_50_ values of 62.53 and 71.67 μM, respectively. All synthesized compounds outperformed Navarixin against HT-29 cells, exhibiting approximately 2.5–3-fold lower IC_50_ values. Similarly, **3a**, **3c**, and especially **3d** demonstrated superior activity against MCF-7 cells, with IC_50_ values reduced by approximately 2–5 fold relative to the reference compound. The most pronounced improvement over Navarixin was observed for IC_50_ of compound **3d** in HL-60 cells, with five-fold increase and six-fold for the selectivity index. Compounds **3a** and **3c** also surpassed Navarixin in A-549 cells, reducing the IC_50_ values from 24.11 μM to 10.33 and 9.65 μM, respectively, while simultaneously increasing selectivity by nearly three-fold. Furthermore, whereas Navarixin displayed only modest selectivity overall (SI = 2.15–6.39), the most active analogues consistently achieved SI values above 10 and, in some cases, above 20, indicating a substantially improved therapeutic profile.

## 3. Materials and Methods

### 3.1. Chemistry

IR spectra were recorded in the range of 4000–400 cm^−1^ on a Bruker Invenio R spectrophotometer (Bruker, Ettlingen, Germany), ATR (attenuated total reflectance) mode with a diamond crystal accessory, 64 scans at a resolution of 2 cm^−1^. The spectra were referenced to air as a background by accumulating 100 scans, at a resolution of 2 cm^−1^. The ^1^H and ^13^C NMR spectra were recorded on an Avance Neo 400 (400 MHz) spectrometer (Bruker, Ettlingen, Germany). Melting points were determined on a Buchi 540 apparatus (BUCHI, Flawil, Switzerland). All of the starting amines were of analytical grade, purchased from Thermo Fisher Scientific (Waltham, MA, USA) and the starting esters **1a** and **1b** were purchased from Fluorochem, Ltd. (Derbyshire, UK). Dimethylsulfoxide-d6 was purchased from Deutero GmbH (Kastellaun, Germany).

#### Synthesis

A solution of compound **1a** or **1b** (0.001 mol) in anhydrous ethanol (5 mL) was prepared in a round-bottom flask equipped with a magnetic stir bar. To this solution, the corresponding amine (0.001 mol) and triethylamine (0.001 mol) were added sequentially. For the squaramides **3e** and **3f**, 2eq. of the amine and triethylamine were used. The reaction mixture was stirred at room temperature for 4–16 h until completion, as monitored by TLC. Upon completion, the reaction mixture was worked up by liquid–liquid extraction using a separatory funnel (DCM/H_2_O), followed by solvent removal under reduced pressure to afford the crude products. They were recrystallized in anhydrous ethanol (5–6 mL.).

**3a:** 3-Methoxy-4-((thiophen-2-ylmethyl)amino)cyclobut-3-ene-1,2-dione. Yield: 78%, m.p.: 107.6–109.9 °C. Isolated as pale-yellow crystals. IR (ATR, cm^−1^) 3141, 2925, 1797, 1691, 1573, 1103.

**3b:** 3-Methoxy-4-((2-(thiophen-2-yl)ethyl)amino)cyclobut-3-ene-1,2-dione. Yield 79% m.p.: 123.1–125.1 °C. Isolated as fine white crystals. IR (ATR, cm^−1^) 3146, 2930, 1802, 1696, 1579, 1095. ^1^H NMR (400 MHz, DMSO-d_6_) δ 8.90 (t, *J* = 6.1 Hz, 0.5H, NH), 8.70 (t, *J* = 6.1 Hz, 0.5H, NH), 7.36 (t, *J* = 5.5 Hz, 1H, thiophene H), 6.97 (m, 1H, thiophene H), 6.89 (m, 1H, thiophene H), 4.26 (d, *J* = 13.7 Hz, 3H, CH_3_), 3.73 (q, *J* = 6.7 Hz, 1H, CH_2_), 3.51 (q, *J* = 6.8 Hz, 1H, CH_2_), 3.06 (q, *J* = 6.4 Hz, 2H, CH_2_). ^13^C-NMR (101 MHz, DMSO-d_6_, δ, ppm) 189.69, 182.86, 177.91, 177.59, 172.87, 172.55, 141.00, 140.60, 127.49, 126.26, 126.11, 125.01, 124.91, 60.59, 60.40, 45.87, 45.26, 31.07, 30.61. 

**3c:** 3-Ethoxy-4-((furan-2-ylmethyl)amino)cyclobut-3-ene-1,2-dione. Yield: 82% m.p.: 95.3–96.2 °C. Isolated as pale-yellow crystals. IR (ATR, cm^−1^) 3146, 3054, 3002, 2922, 1803, 1645, 1554. ^1^H NMR (400 MHz, DMSO-d_6_) δ 9.24 (s, 0.5H, NH), 9.04 (s, 0.5H, NH), 7.64 (s, 1H, furan H), 6.43 (s, 1H, furan H), 6.34 (d, *J* = 3.1 Hz, 1H, furan H), 4.67 (m, 3H, CH_2_) 4.47 (d, *J* = 6.1 Hz, 1H, CH_2_), 1.37 (t, *J* = 7.1 Hz, 3H, CH_3_) ^13^C-NMR (101 MHz, DMSO-d_6_, δ, ppm) 189.67, 183.02, 177.49, 173.25, 151.31, 143.46, 111.13, 108.42, 69.43, 51.16, 16.09. 

**3d:** 3,4-Bis((thiophen-2-ylmethyl)amino)cyclobut-3-ene-1,2-dione. Yield: 70% m.p.:257.8–259.1 °C Isolated as white amorphous solid. IR (ATR, cm^−1^) 3145, 3070, 2999, 2926, 1798, 1691, 1645, 1562. ^1^H NMR (400 MHz, DMSO-d_6_) δ 7.81 (s, 2H, NH), 7.47 (dd, *J* = 5.0, 1.3 Hz, 2H, thiophene H), 7.04 (d, *J* = 2.3 Hz, 2H, thiophene H), 7.00 (dd, *J* = 5.1, 3.4 Hz, 2H, thiophene H), 4.89 (d, *J* = 6.3 Hz, 4H, CH_2_). ^13^C-NMR (101 MHz, DMSO-d_6_, δ, ppm) 183.15, 167.71, 142.17, 127.58, 126.60, 126.46, 42.07.

**3e:** 3,4-Bis((furan-2-ylmethyl)amino)cyclobut-3-ene-1,2-dione. Yield: 81% m.p.:242.1–245.1 °C Isolated as white amorphous solid. IR (ATR, cm^−1^) 3146,3120,3054,3004,2921,1803,1645,1559. ^1^H NMR (400 MHz, DMSO-d_6_) δ 7.68 (s, 2H, NH), 7.64 (dd, *J* = 1.9, 0.9 Hz, 2H, furan H), 6.43 (dd, *J* = 3.2, 1.8 Hz, 2H, furan H), 6.35 (d, *J* = 3.2 Hz, 2H, furan H), 4.73 (d, *J* = 6.1 Hz, 4H, CH_2_). ^13^C-NMR (101 MHz, DMSO-d_6_, δ, ppm) 183.17, 167.95, 152.16, 143.37, 111.10, 108.17, 40.31.

### 3.2. Density Functional Theory (DFT) Study

The structural and vibrational characteristics were analyzed by DFT calculations using the Gaussian 09 software. The computations were done employing the B3LYP (Becke, 3-parameter, Lee-Yang-Parr) and 6-311++G** basis set [38,39,40]. 

### 3.3. In-Silico Modeling

The molecular structures of the investigated compounds were constructed and visualized using the ACD/Labs Suite v. 9.10 (Advanced Chemistry Development, Inc., Toronto, ON, Canada). Relevant molecular descriptors employed in the development of QSPR models were subsequently computed using the MDL QSAR software (version 2.2.0.0.446; MDL Information Systems, Inc., San Leandro, CA, USA, 2004).

### 3.4. Single-Crystal X-Ray Diffraction (SCXRD) Analysis

Single crystals with suitable quality of **3a**–**3c** are fished on a nylon loop. The diffraction data for the compounds is collected on a Bruker D8 Venture diffractometer (Bruker AXS GmbH, Karlsruhe, Germany) equipped with a PhotonII CMOS detector (Bruker, Karlsruhe, Germany) using micro-focus MoK_ radiation (λ = 0.71073 Å). Data reduction is performed with APEX4 software (version 2021.4-5) [41]. The structures are solved with intrinsic methods using ShelxT and refined by the full-matrix least-squares method on *F*^2^ with SHELXL-2018/3 program. All non-hydrogen atoms are located successfully from Fourier map and are refined anisotropically. Hydrogen atoms are placed on calculated positions (C–H_methyl_ = 0.96 Å, C–H_aromatic_ = 0.93 Å and C–H_methylenic_ = 0.97 Å) riding on the parent atom (Ueq = 1.2). Most important data collection and crystallographic refinement parameters of **3a**–**3c** are provided in the Supplementary Material. Complete crystallographic data for the structures of **3a**–**3c** reported in this paper has been deposited in CIF format with the Cambridge Crystallographic Data Center. These data can be obtained free of charge via https://www.ccdc.cam.ac.uk/structures/ (accessed on 3 February 2026) or from the CCDC, 12 Union Road, Cambridge CB2 1EZ, UK; Fax: +44-12-2333-6033; E-mail: deposit@ccdc.cam.ac.uk).

### 3.5. In Vitro Assessment of Cytotoxicity

Human cancer cell lines HL-60 (acute myeloid leukemia, DSMZ No. ACC 3), A-549 (lung carcinoma, DSMZ No. ACC 107), HT-29 (colon adenocarcinoma, DSMZ No. ACC 299), MCF-7 (breast adenocarcinoma, DSMZ No. ACC 115), and the non-cancerous CCL-1 (murine fibroblasts, DSMZ No. ACC 2) were obtained from the German Collection of Microorganisms and Cell Cultures (Braunschweig, Germany). Cells were maintained in Dulbecco’s Modified Eagle Medium (DMEM; Thermo Fisher Scientific, Waltham, MA, USA) supplemented with 10% fetal bovine serum (FBS) and horse serum for the CCL-1 line, 1% L-glutamine, and 1% penicillin/streptomycin in a humidified incubator at 37 °C with 5% CO_2_. Adherent cell lines (A-549, HT-29, MCF-7, HeLa, and CCL-1) were routinely passaged at 85–90% confluence using trypsin-EDTA, while suspension HL-60 cells were maintained by dilution. For cytotoxicity evaluation, cells in the exponential growth phase were seeded in 96-well plates (100 µL per well) at a density of 3 × 10^5^ cells/well for HL-60 and 2.5 × 10^5^ cells/well for the adherent cell lines. After 24 h incubation, they were treated with six serial dilutions of the compounds **3a**–**3e** over an appropriate concentration range. Following 72 h exposure, 10 µL of sterile MTT solution (10 mg/mL in PBS) was added to each well and incubated for an additional 3 h to allow the formation of formazan crystals. The resulting crystals were dissolved in isopropanol containing 5% formic acid, and absorbance was measured at 550 nm using a microplate reader Labexim LMR-1 (Labexim Products, Vienna, Austria) [42,43]. Cell viability was calculated by normalizing absorbance values to untreated controls (defined as 100%). IC_50_ were determined by nonlinear regression analysis using GraphPad Prism 9 software for Windows, based on semi-logarithmic dose-response curves.

## 4. Conclusions

In this study, a series of new squaramides and squaramates incorporating five-membered heterocycles was successfully synthesized and characterized. The IR spectra showed characteristic carbonyl bands at ≈1800 and ≈1700 cm^−1^, while amino stretching appeared at ≈3145 cm^−1^, consistent with hydrogen bonding. The ^1^H NMR spectra confirmed the structures, with N-H signals at 8.5–9.5 ppm and heteroaromatic protons in the 6–8 ppm region. X-ray and DFT analyses were in good agreement, with only minor deviations, particularly for N-H vibrations due to H-bonding effects. In silico evaluation indicated favorable drug-like properties with all compounds complying with Lipinski’s rule and showing high predicted gastrointestinal absorption. Cytotoxicity study, via MTT test revealed that compounds **3a** and **3c** have exceptional activity against A-549 cells (IC_50_ = 10.33 and 9.65 µM; SI ≈ 17.7 and 19.7), whereas compound **3d** emerged as the most promising analogue due to its outstanding potency and selectivity against HL-60 leukemia cells (IC_50_ = 5.81 µM; SI ≈ 29.1) and its superior overall performance compared with the reference compound. Moderate activity was also observed against MCF-7 cells. All compounds exhibited low toxicity toward non-cancerous CCL-1 cells (IC_50_ > 160 µM). In comparison, Navarixin showed 2–5-fold weaker cytotoxicity amongst the series and a lower cancer selectivity index. Taken together, these results demonstrate that structural modification of the Navarixin molecule led to derivatives with enhanced cytotoxic potency and improved selectivity toward several cancer cell lines, highlighting SQ derivatives as promising anticancer scaffolds and providing a solid basis for further optimization and mechanistic studies.

## Figures and Tables

**Table 1:** Structures of the synthesized derivatives of squaric acid.

Structure	ID	R1	R2
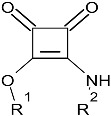	**3a**	Methyl-	Thiophen-2-ylmethyl-
	**3b**	Methyl-	Thiophen-2-ylethyl-
	**3c**	Ethyl-	Furan-2-ylmethyl-
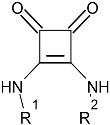	**3d**	Thiophen-2-ylmethyl-	Thiophen-2-ylmethyl-
	**3e**	**Furan-2ylmethyl-**	**Furan-2ylmethyl-**

**Table 2:** Estimated Physicochemical and Pharmacokinetic Profiles of SQ derivatives.

Property	3a	3b	3c	3d	3e
**Mw g/mol**	223.25	237.27	221.21	304.38	272.26
**pK_a_**	−2.01	−0.65	−1.84	−0.76	−0.53
**f_b_**	0	0	0	0	0
**logP**	1.13	1.40	0.84	2.24	0.89
**PSA Å^2^**	83.64	83.64	68.54	114.68	84,48
**FRB**	4	5	5	6	6
**HBAs**	3	3	4	2	4
**HBDs**	1	1	1	2	2
**R5**	0	0	0	0	0
**S**	Soluble	Soluble	Very soluble	Moderately soluble	Soluble
**GI absorp**	High	High	High	High	High
**Oral BA**	INSATU	SATU	SATU	INSATU	INSATU
**BA score**	0.56	0.56	0.85	0.55	0.55
**BBB permeab**	No	No	No	No	No
**CYP inhib**	1A2	1A2, 2C19	1A2	1A2, 2C19, 2C9, 3A4	1A2
**P-gp substr**	No	No	No	No	No
**Drug likeness**	Yes	Yes	Yes	Yes	Yes
**Lead likeness**	Yes *	Yes *	Yes *	Yes	Yes
**SA**	2.58	2.72	2.80	2.87	2.94
**f_u_**	0.230	0.188	0.257	0.084	0.138
**CL mL/min/kg**	4.541	4.050	5.076	3.699	4.425
**VDss L/kg**	1.008	1.057	0.827	2.135	1.578
**t_1/2_ h**	2.59	3.05	1.91	6.73	4.21

Mw—molecular weight, pKa value, f_b_—fraction of the ionized molecules at pH 7.4, logP—partition coefficient, PSA—polar surface area, FRB—count of free rotatable bonds, HBAs—hydrogen bond acceptors, HBDs—hydrogen bond donors, R5—count of the violations from Lipinski’s Rule of 5, S—water solubility, GI absorp—gastrointestinal absorption, Oral BA—oral bioavailability, INSATU—unsaturation, BA score—bioavailability score, BBB permeab—blood–brain barrier permeability, CYP inhib—inhibition of CYP enzymes, P-gp substr—substrate of P-gp, drug likeness, lead likeness, SA—synthetic accessibility, f_u_—fraction of the unbound to plasma protein molecules, CL—total clearance, VDss—steady-state volume of distribution; t_1/2_—half-life; * Mw < 250 g/mol.

**Table 3:** Selected theoretical and experimental bond lengths and angles for **3a**–**3e**.

Geometric Parameters	3a	3a	3b	3b	3c	3c	3d	3d	3e	3e
	DFT	X-Ray	DFT	X-Ray	DFT	X-Ray	DFT	X-Ray	DFT	X-Ray
**Bond lengths (Å)**										
C3–N7	1.33	1.30	1.33	1.31	1.33	1.32	1.34	–	1.34	–
C3–C4	1.39	1.39	1.39	1.39	1.39	1.38	1.39	–	1.39	–
C4–Y9	1.32	1.31	1.32	1.32	1.32	1.30	1.34	–	1.34	–
**Angles (°)**										
C3–N7–R8	123.8	122.4	123.3	122.0	122.2	122.7	123.1	–	121.0	–
C4–Y9–R10	116.4	116.0	116.4	115.5	117.0	116.7	123.0	–	121.0	–

**Table 4:** Cytotoxic activity expressed as IC_50_ (µM); SI values are given in parentheses.

Cell Line	3a	3b	3c	3d	3e	Nav
HeLa	>100	>100	>100	>100	>100	>100
HT-29	61.42 (2.98)	20.42 (9.66)	24.95 (7.63)	20.44 (8.27)	22.70 (8.14)	62.53 (2.46)
HL-60	42.14 (4.35)	70.68 (2.79)	26.78 (7.11)	5.81 (29.07)	74.47 (2.48)	29.10 (5.30)
A-549	10.33 (17.73)	36.40 (5.42)	9.65 (19.73)	34.47 (4.90)	23.63 (7.82)	24.11 (6.39)
MCF-7	23.87 (7.67)	40.09 (4.92)	34.26 (5.56)	15.74 (10.73)	39.31 (4.70)	71.67 (2.15)
CCL-1	183.18	197.33	190.38	168.90	184.70	154.26

## Data Availability

The raw data supporting the conclusions of this article will be made available by the authors on request.

## Supplementary Materials

The following supporting information can be downloaded at: https://www.mdpi.com/article/10.3390/ijms27136047/s1.

## Abbreviations

The following abbreviations are used in this manuscript:

SQ	squaric acid
DFT	density functional theory
Nav	Navarixin
ATR	Attenuated Total Reflectance
