# Supplementary material for: Novel Squaramides and Squaramates Containing a Five-Membered Heterocyclic Ring: Synthesis, Structure, and Cytotoxicity
Source: Int J Mol Sci. 2026 Jul 6;27(13):6047. doi: 10.3390/ijms27136047 (PMC13361079; doi:10.3390/ijms27136047)
Supplement: Supplementary file 1 [file ijms-27-06047-s001.zip › ijms-4400675-supplementary.pdf]

Novel Squaramides and Squaramates Containing a Five-Membered Heterocyclic Ring: Synthesis, Structure and Cytotoxicity

Georgi Tirolski[1,2], Boris Vasilev[1], Mariyana Atanasova[3,4], Georgi Momekov[1], Hristina Sbirkova-Dimitrova[5,6], Adriana Bakalova[3*], Emiliya Cherneva[3,4*]

[1] Department of Pharmacology, Pharmacotherapy and Toxicology, Faculty of Pharmacy, Medical University of Sofia, Dunav -2 Street, 1000, Sofia, Bulgaria
[2] Institute of Organic Chemistry with Centre of Phytochemistry, Bulgarian Academy of Sciences, Acad. G. Bonchev Str., Build. 9, 1113, Sofia, Bulgaria
[3] Department of Chemistry, Faculty of Pharmacy, Medical University of Sofia, Dunav -2 Street, 1000, Sofia, Bulgaria
[4] Centre of Excellence in Informatics and Information and Communication Technologies, 1113 Sofia, Bulgaria
[5] Institute of Mineralogy and Crystallography "Acad. Ivan Kostov" - Bulgarian Academy of Sciences, Acad. G Bonchev sstr. Bl. 107, Sofia 1113, Bulgaria
[6] Center of Competence -PERIMED-2, Vasil Aprilov Blvd. 15A, 4002 Plovdiv, Bulgaria
*Correspondence: a_bakalova@pharmfac.mu-sofia.bg (A.B); echerneva@pharmfac.mu-sofia.bg (E.Ch.)

Contents

Table S1. Crystal data and structure refinement parameters for compounds 3a–c.
Scheme S1. Crystal packing and intermolecular hydrogen-bonding interactions in compounds **3a-**c determined by X-ray crystallographic analysis. Hydrogen bonds are shown as dashed lines.
Scheme S2. Optimized geometries and relative Gibbs free energies (ΔG, kJ/mol) of the most stable conformers of compounds **3a-e** calculated at the B3LYP/6-311++G** level of theory.
Figure S1. IR-ATR spectrum (4000–400 cm^{-1}) of compound **3a**.
Figure S2. IR-ATR spectrum (4000–400 cm^{-1}) of compound **3b**.
Figure S3. IR-ATR spectrum (4000–400 cm^{-1}) of compound **3c**.
Figure S4. IR-ATR spectrum (4000–400 cm^{-1}) of compound **3d**.
Figure S5. IR-ATR spectrum (4000–400 cm^{-1}) of compound **3e**.
Figure S6. ^{1}H NMR spectrum of compound **3b**.
Figure S7. ^{13}C NMR spectrum of compound **3b**.
Figure S8. ^{1}H NMR spectrum of compound **3c**.
Figure S9. ^{13}C NMR spectrum of compound **3c**.
Figure S10. ^{1}H NMR spectrum of compound **3d**.
Figure S11. ^{13}C NMR spectrum of compound **3d**.
Figure S12. ^{1}H NMR spectrum of compound **3e**.
Figure S13. ^{13}C NMR spectrum of compound **3e**.

Table S1 Crystal data and structure refinement for **3a**, **3b** and **3c**

**Identification code**	**3a**	**3b**	**3c**
**Empirical formula**	$C_{10}H_9NO_3S$	$C_{11}H_{11}NO_3S$	$C_{11}H_{11}NO_4$
**Formula weight**	223.24	237.27	221.21
**Temperature/K**	293.00	295.00	273.15
**Crystal system**	monoclinic	monoclinic	triclinic
**Space group**	*P*c	$P2_1$/n	*P*-1
***a*/Å**	6.6569(7)	12.1230(4)	6.368(2)
***b*/Å**	18.5277(16)	6.7076(3)	8.457(3)
***c*/Å**	8.6411(8)	13.9688(5)	10.633(4)
**α/°**	90	90	96.910(11)
**β/°**	99.082(3)	96.8780(10)	98.510(11)
**γ/°**	90	90	98.570(12)
**Volume/Å³**	1052.41(17)	1127.72(7)	554.0(3)
**Z**	4	4	2
ρ_{calc}**g/cm³**	1.409	1.397	1.326
**μ/mm^{-1}**	0.293	0.278	0.102
**F(000)**	464.0	496.0	232.0
**Crystal size/mm³**	0.18×0.16 ×0.15	0.22×0.18×0.15	0.15×0.15×0.12
**Radiation**	Mo*Kα* (*λ* = 0.71073)	Mo*Kα* (*λ* = 0.71073)	Mo*Kα* (*λ* = 0.71073)
**2Θ range for data collection/°**	4.396 to 52.776	4.206 to 52.756	5.828 to 53.068
**Reflections collected**	29742	30297	26191
R_{int} /R_{sigma}	0.0320/0.0278	0.0699/0.0303	0.0483/0.0300
**Data/restraints/parameters**	4320/145/327	2305/45/170	2272/0/146
**Goodness-of-fit on F²**	1.068	1.034	1.106
**Final *R* indexes [I>=2σ (I)]**	R_1= 0.0364 wR_2= 0.1022	R_1= 0.0650 wR_2= 0.1899	R_1= 0.0785 wR_2= 0.1975
**Final *R* indexes [all data]**	R_1= 0.0374, wR_2= 0.1034	R_1= 0.0752 wR_2= 0.2012	R_1= 0.0912 wR_2= 0.2067
**Largest diff. peak/hole / e Å^{-3}**	0.17/-0.17	0.42/-0.47	0.45/-0.24
**Flack parameter**	0.060(17)	-	-
**CCDC number**	2539468	2539469	2539470

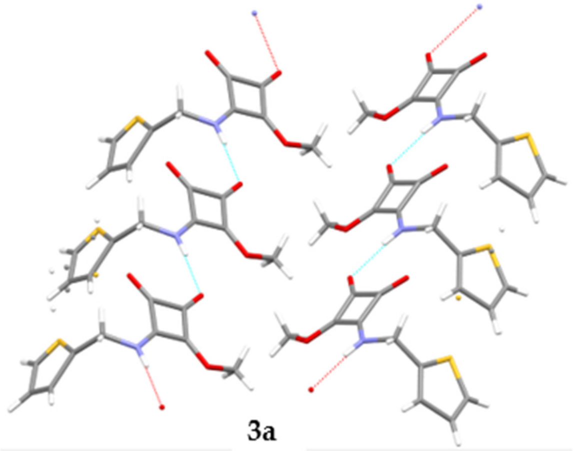

3a

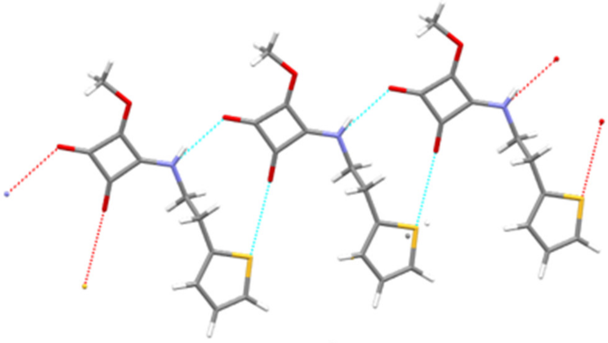

3b

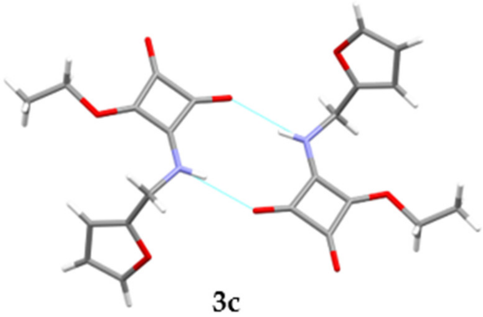

3c

Scheme S1 Crystal packing and intermolecular hydrogen-bonding interactions in compounds **3a-c** determined by X-ray crystallographic analysis. Hydrogen bonds are shown as dashed lines.

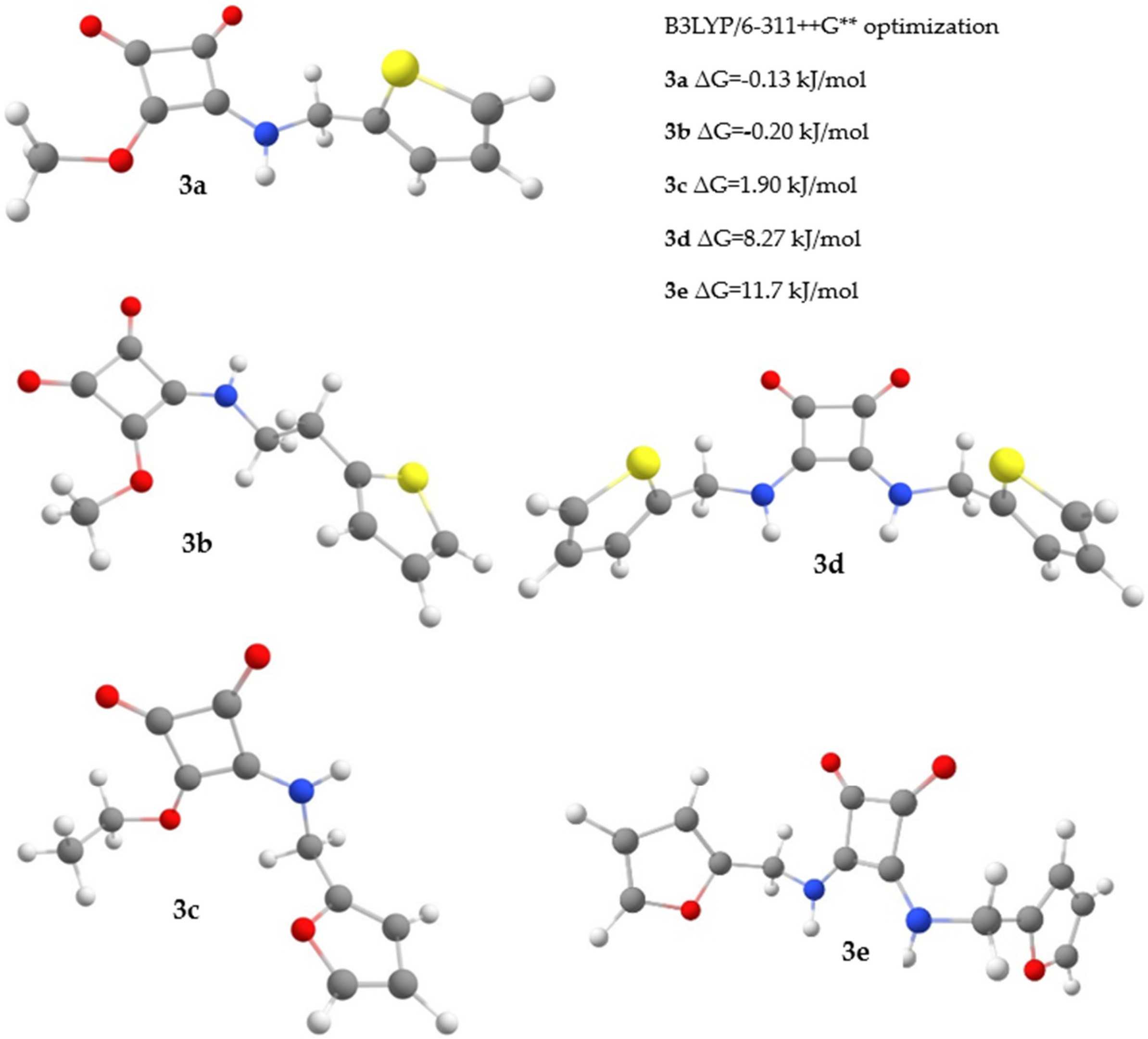

Scheme S2 Optimized geometries and relative Gibbs free energies (ΔG, kJ/mol) of the most stable conformers of compounds **3a-e** calculated at the B3LYP/6-311++G** level of theory.

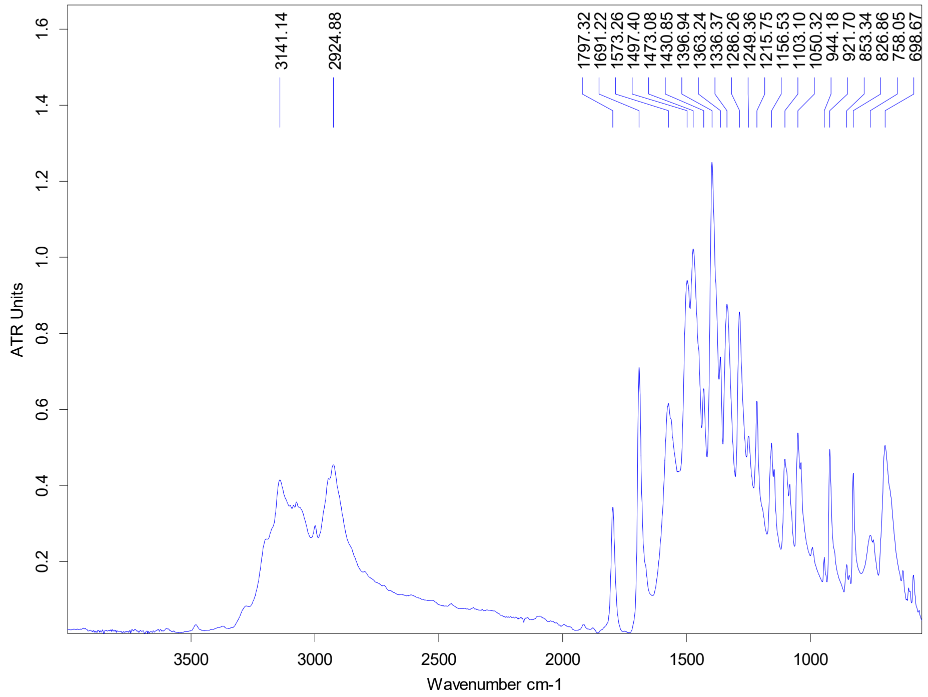

Figure S1 IR-ATR spectrum at 4000-400 cm^{-1} of **3a**

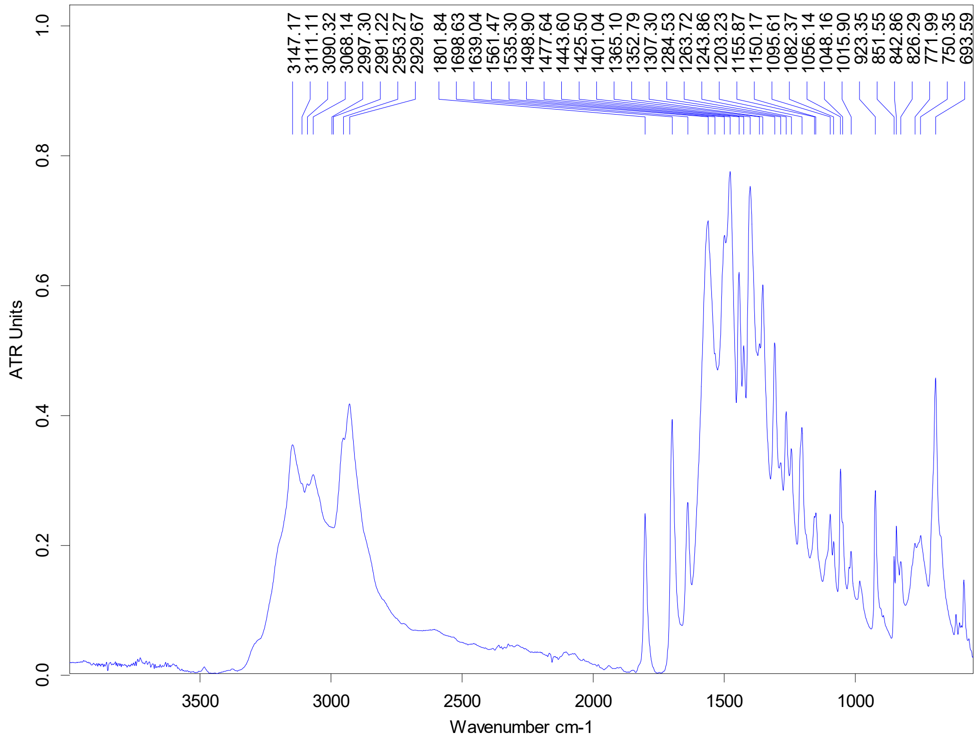

Figure S2 IR-ATR spectrum at 4000-400 cm^{-1} of **3b**

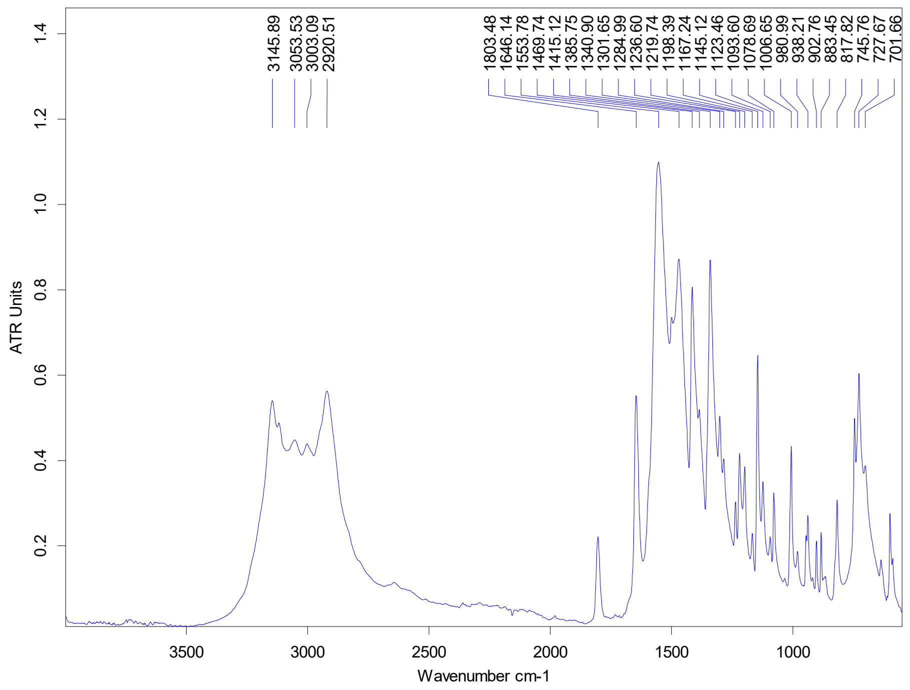

Figure S3 IR-ATR spectrum at 4000-400 cm^{-1} of **3c**

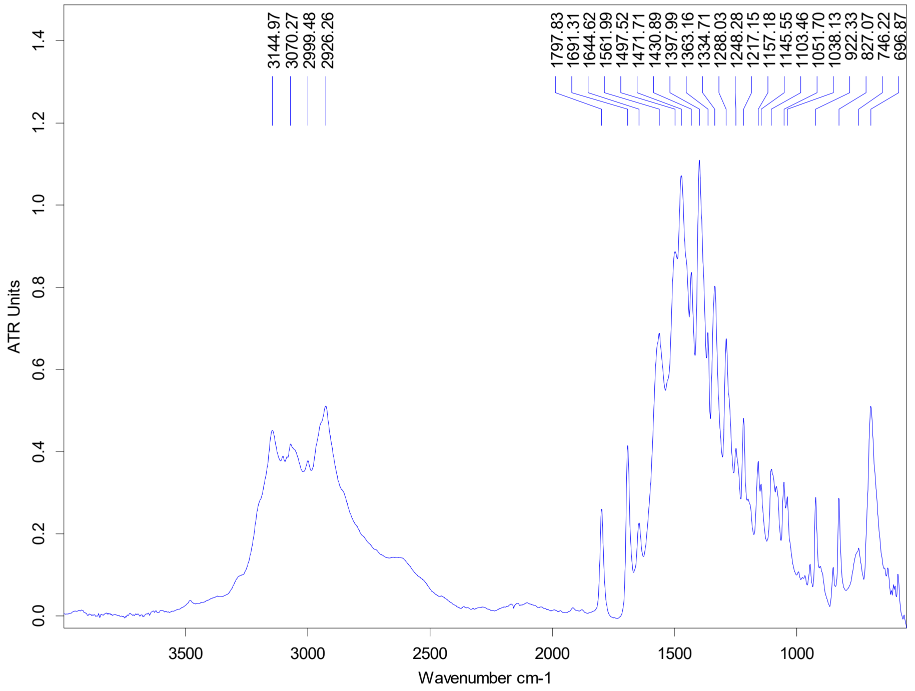

Figure S4 IR-ATR spectrum at 4000-400 cm^{-1} of **3d**

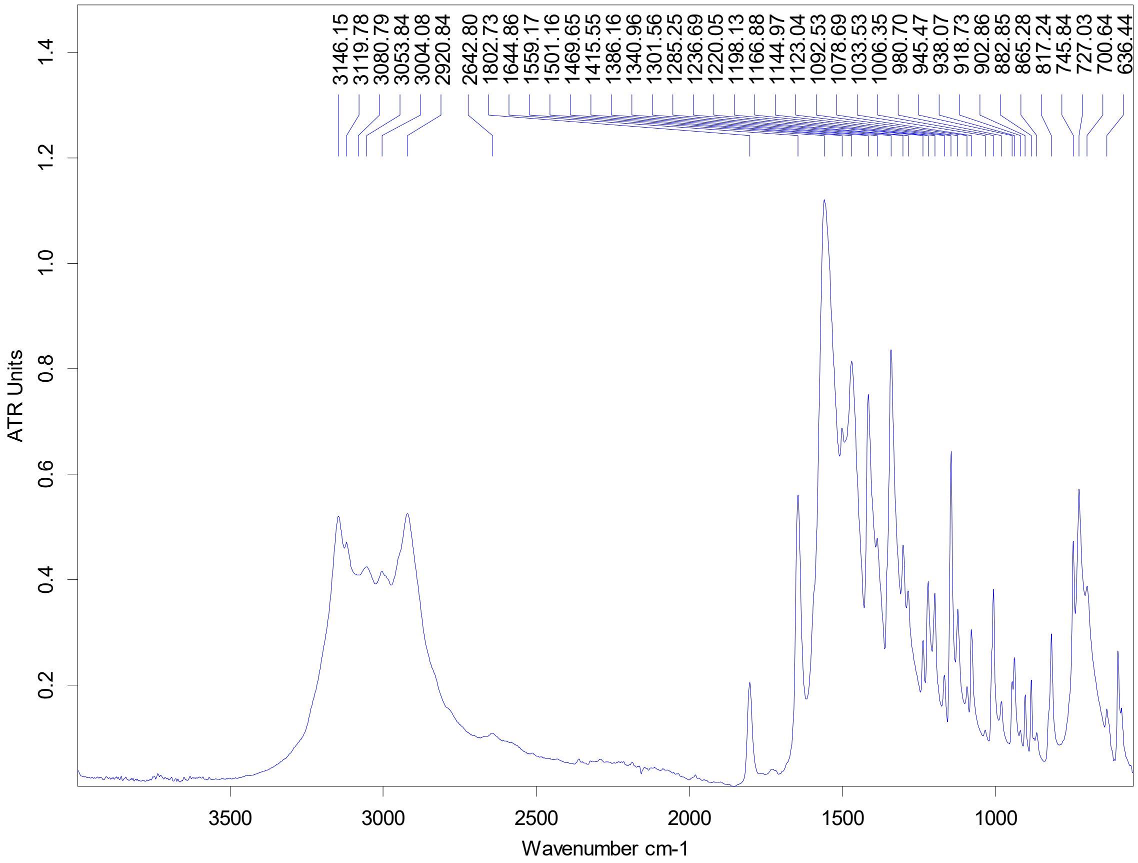

Figure S5 IR-ATR spectrum at 4000-400 cm^{-1} of **3e**

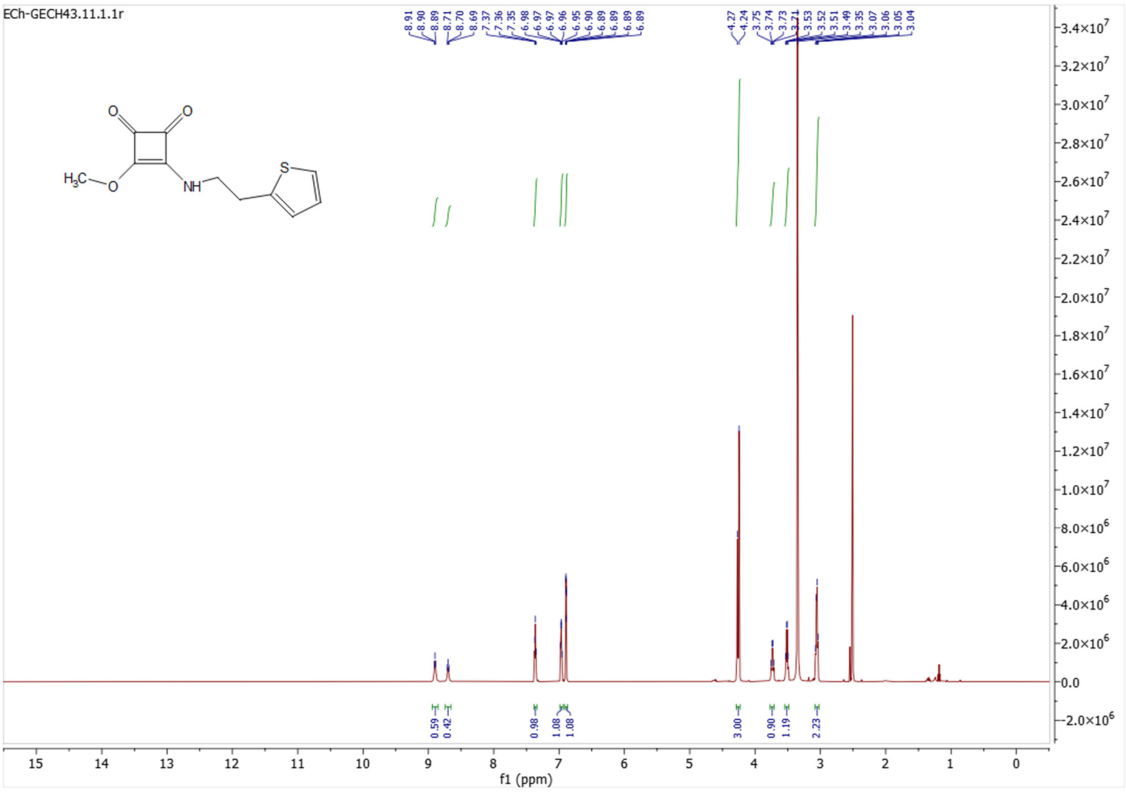

Figure S6 ^{1}H NMR spectrum of **3b**

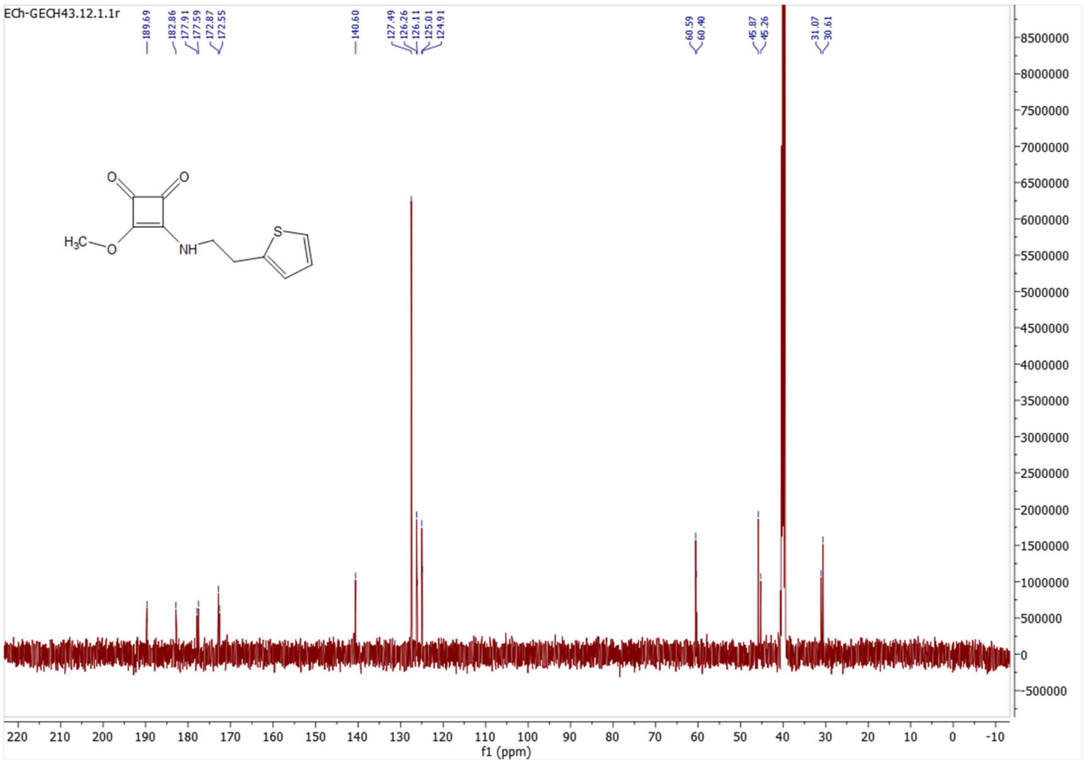

Figure S7 ^{13}C NMR spectrum of **3b**

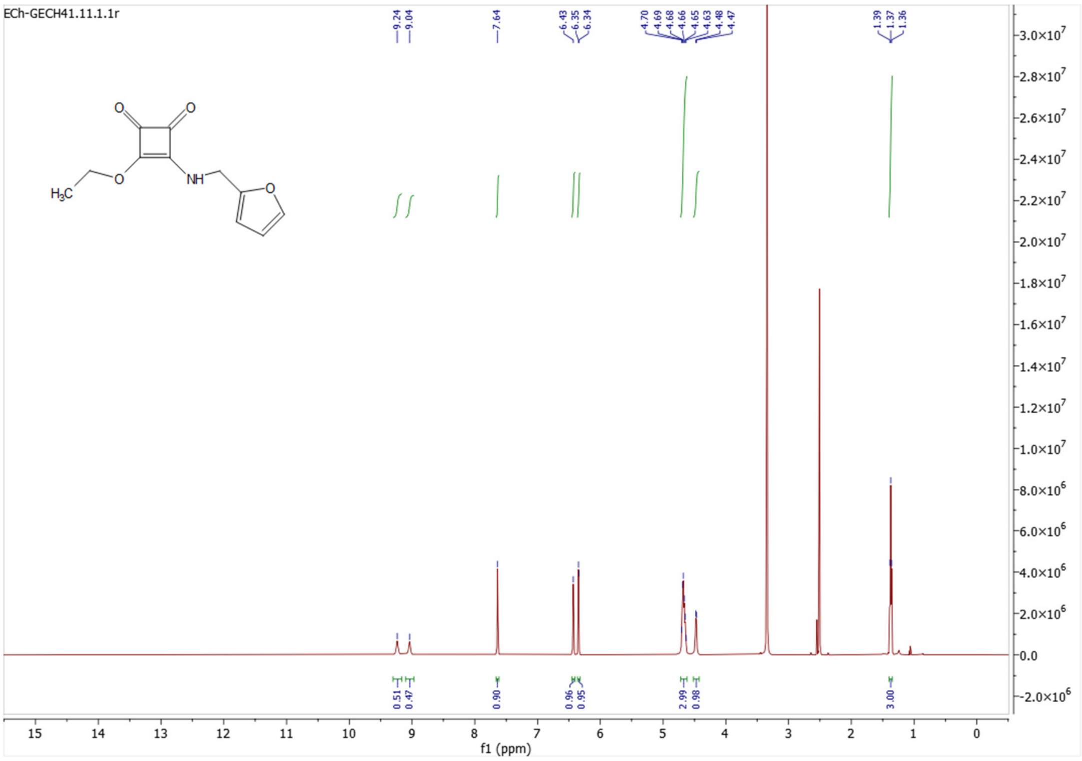

Figure S8 ^{1}H NMR spectrum of **3c**

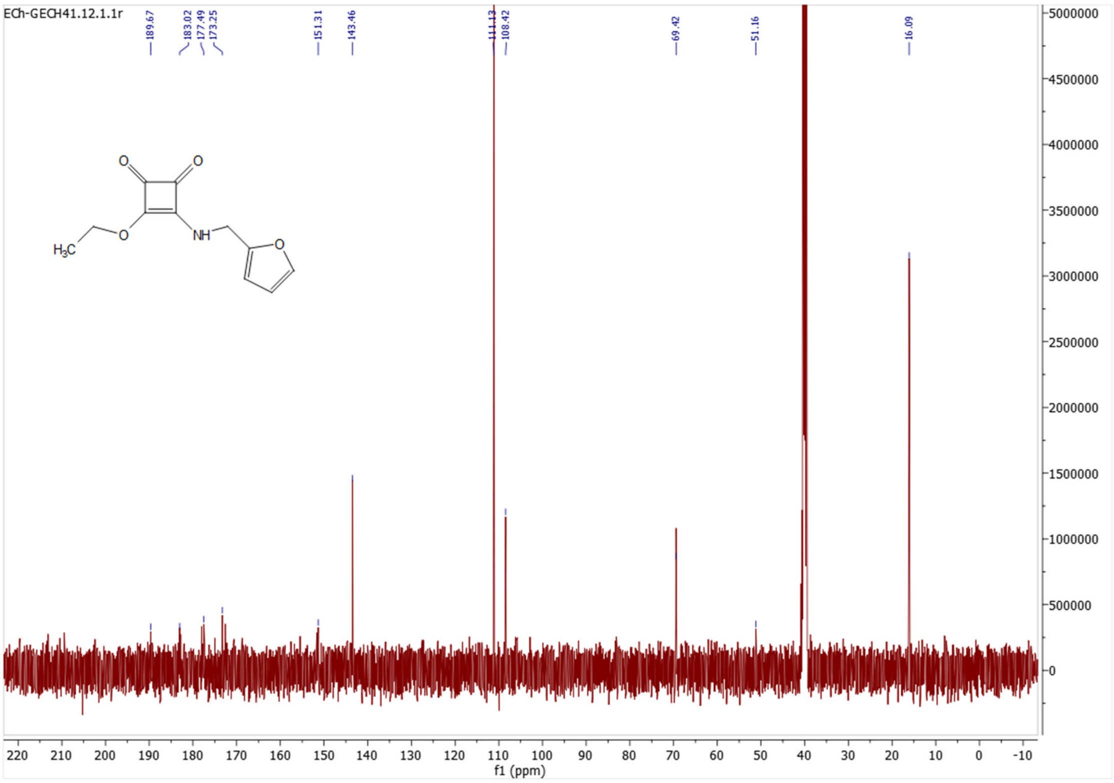

Figure S9 ^{13}C NMR spectrum of **3c**

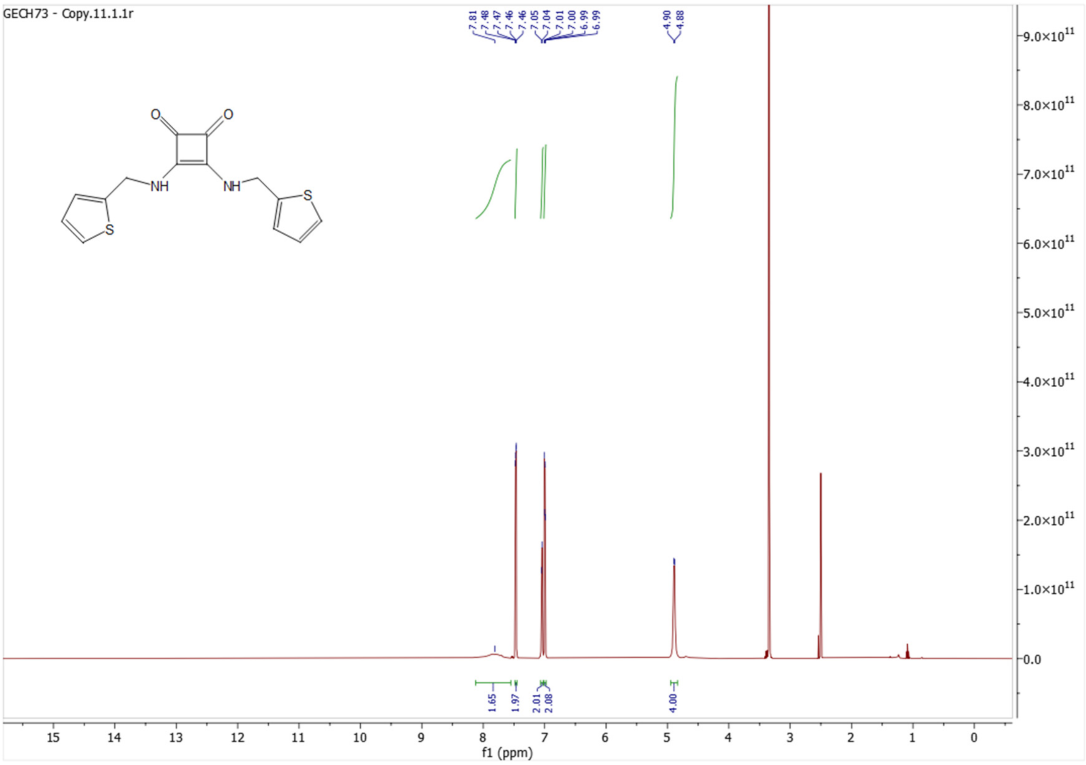

Figure S10 ^{1}H NMR spectrum of **3d**

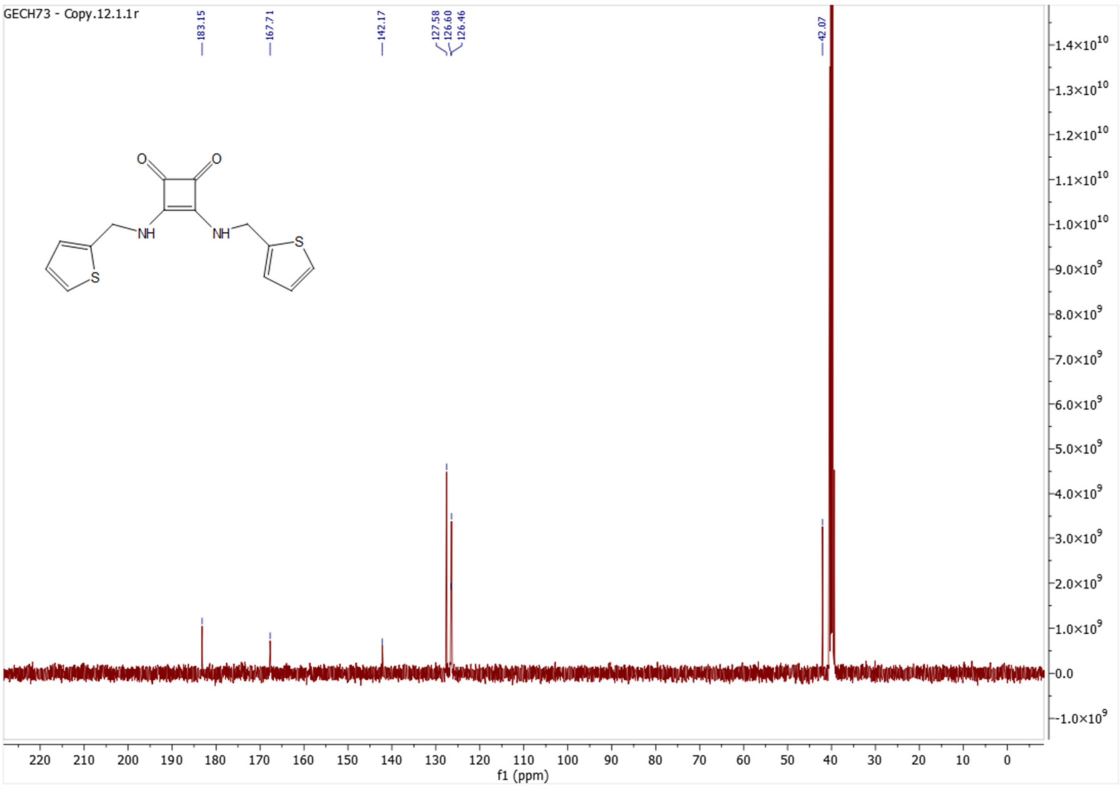

Figure S11 ^{13}C NMR spectrum of **3d**

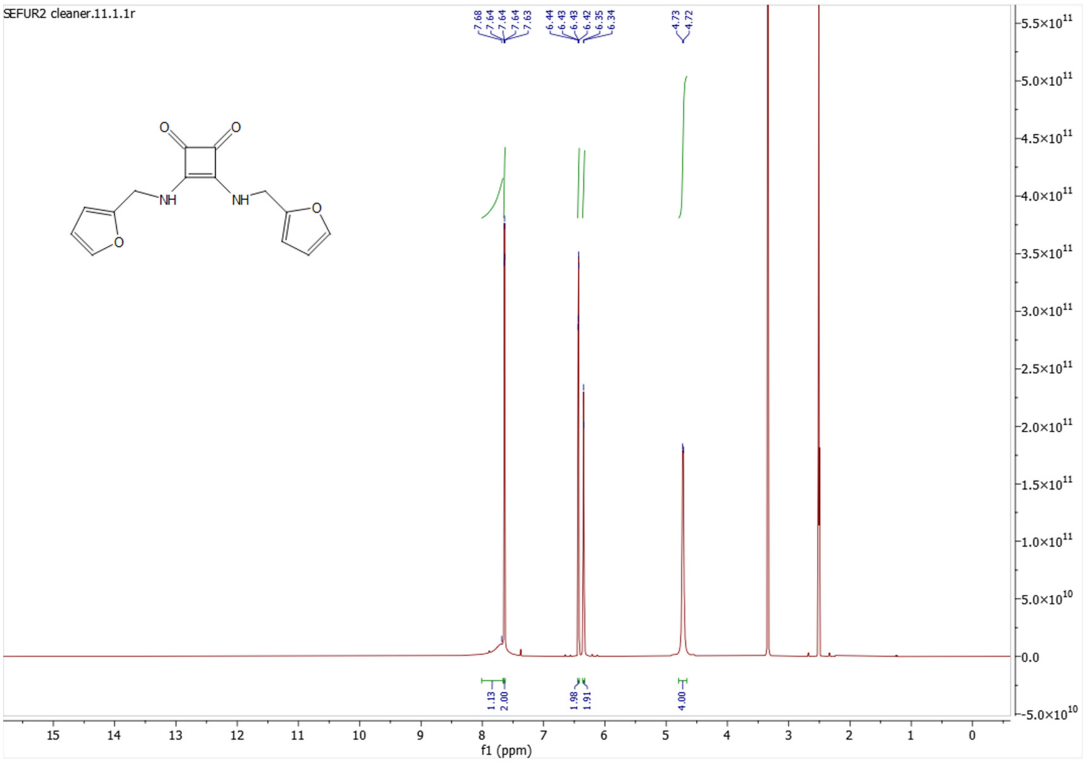

Figure S12 ¹H NMR spectrum of **3e**

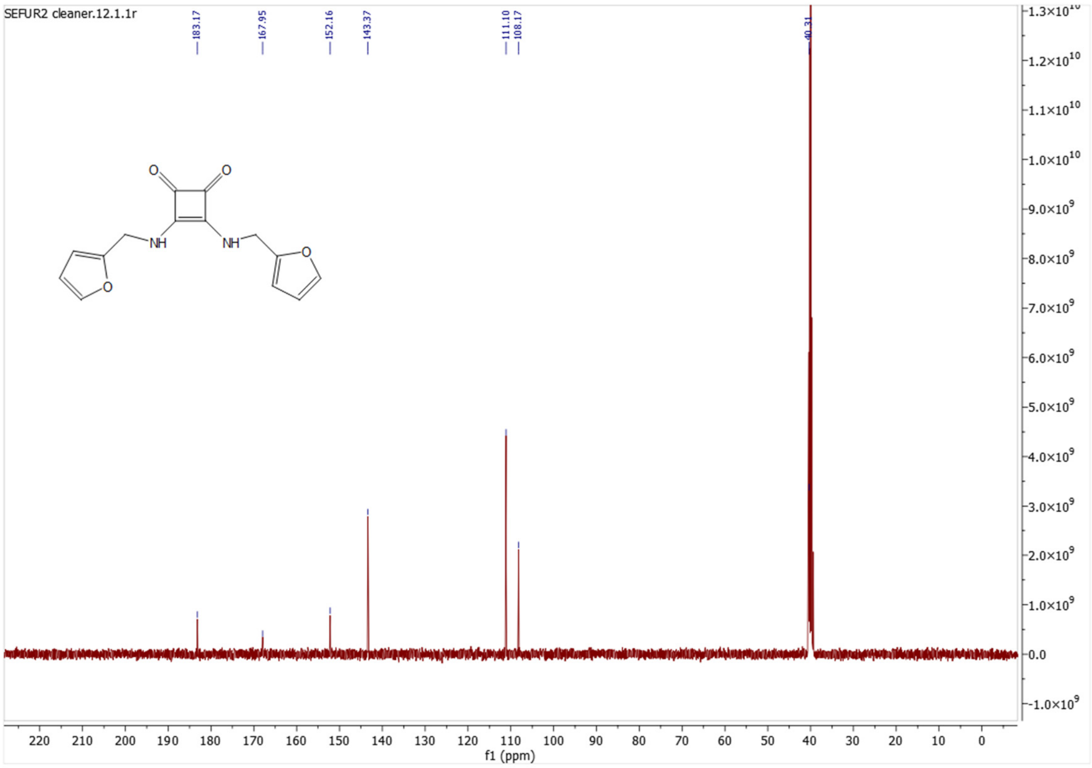

Figure S13 ¹³C NMR spectrum of **3e**
